# Oral-History-Projekt Humangenetik: Historische Forschungsmethode zur Erhebung und Weiterverarbeitung narrativer Interviews

**DOI:** 10.1515/medgen-2021-2078

**Published:** 2021-08-14

**Authors:** F. Söhner, H. Fangerau, M. Krischel

**Affiliations:** Institut für Geschichte, Theorie und Ethik der Medizin, Centre for Health and Society, Medizinische Fakultät, Heinrich-Heine-Universität Düsseldorf, Moorenstraße 5, 40225 Düsseldorf, Deutschland

**Keywords:** Medizinische Genetik, Medizingeschichte, Oral History, Zeitgeschichte, Deutsche Gesellschaft für Humangenetik, medical genetics, history of medicine, oral history, contemporary history, German society for human genetics

## Abstract

Zwischen 2016 und 2018 wurden mit 33 Personen Interviews zur Geschichte der Humangenetik in Deutschland zwischen 1970 und den 2000er Jahren geführt. 29 Interviewte stimmten einer wissenschaftlichen Analyse zu. Diese Interviews wurden mit den Methoden der qualitativen Inhaltsanalyse und der Grounded Theory ausgewertet. Im Zentrum dieses Beitrags steht die kritische Auseinandersetzung mit der Methode der Oral History und ihrer Anwendung auf die Humangenetik. Das Oral-History-Projekt konzentriert sich auf Fragen zu (1) biographischen Daten und Werdegang der Gesprächspartner*innen, (2) Entwicklung und Anwendung von diagnostischen und therapeutischen Techniken, (3) Etablierung und Ausbau der Institutionen der Humangenetik und (4) der Wahrnehmung der das Fach betreffenden gesellschaftlichen Debatten.

## Abstract (en)

Between 2016 and 2018, interviews with 33 persons were conducted about the history of human genetics in Germany between 1970 and the 2000s. 29 interviewees gave consent to have the interviews used for historical research. These interviews are currently being analysed with the methods of qualitative content analysis and grounded theory. The focus of this article lies on the critical examination of the method of oral history and its application to human genetics. The oral history project focuses on questions regarding (1) biographical data and careers of interviewees, (2) development and application of diagnostic and therapeutic techniques, (3) establishment and growth of institutions of human genetics and (4) social debates regarding the discipline.

Wichtige professionspolitische und technische Entwicklungen der Humangenetik in Deutschland fallen in die Zeitgeschichte. In der jüngsten Vergangenheit sind das die Einführung der Humangenetik als eigenes Fachgebiet im Jahr 1992 und die Gründung der Deutschen Gesellschaft für Humangenetik (GfH) im Jahr 1987. Etwas weiter zurück liegen etwa die Einführung der Zusatzbezeichnung Medizinische Genetik im Jahr 1978 [1] sowie die Einführung der Amniozentese [2] und der genetischen Beratung [3] in die klinische Versorgung in den 1970er Jahren.

Den Bogen von einer Wissenschaftsgeschichte zu einer Sozialgeschichte der Humangenetik in Deutschland zwischen ca. 1970 und den 2000er Jahren spannt ein 2016 von der GfH initiiertes Forschungsprojekt. [4] Dessen Ziel ist es, die Entwicklung der (bundes-)deutschen Humangenetik in ihrem Selbstverständnis „als Querschnittsfach (Verzahnung mit nahezu allen klinischen Fächern) und als Längsschnittfach (Brückenbildung von der Grundlagenforschung bis zum Patienten in der Genetischen Beratung)“ [5] ebenso zu dokumentieren und zu reflektieren wie die soziale Einbettung des Fachs. Eine zentrale Rolle nehmen im Projekt Interviews mit fachlichen Akteur*innen ein, deren Erinnerungen dokumentiert und an anderen historischen Quellen gespiegelt werden.

Wichtigstes Anliegen dieses Oral-History-Projekts ist es, die Stimmen von Akteur*innen mit humangenetischer Qualifikation,1Dazu zählen: Facharzt für Humangenetik, Zusatzbezeichnung „Medizinische Genetik“ oder „Fachhumangenetiker GfH/GAH“. (vgl. Berufsverband Medizinische Genetik e. V., Deutsche Gesellschaft für Humangenetik, Leitlinien zur Erbringung humangenetischer Leistungen: 1. Leitlinien zur Genetischen Beratung, medgen. 1996(8): Heft 3 (Sonderbeilage): 1–2). die an diesen Entwicklungen wesentlich beteiligt waren, dauerhaft zu bewahren. Im Folgenden werden der Wert der „Oral History“ und damit der Interviews mit Humangenetiker*innen für die Zeitgeschichte, die Auswahl der Gesprächspartner, die Interviewdurchführung und Auswertungsstrategien vorgestellt. Zuletzt wird ein Ausblick auf erste Ergebnisse gegeben.

## Oral History als Zugang zu Erinnerung

Biographische Interviews bieten in der Geschichtswissenschaft unter der Bezeichnung „Oral History“ einen Ansatz, der eine qualitative Erschließung und Analyse von einzelnen Perspektiven auf Geschichte beabsichtigt. [6] Eine Stärke der Oral History liegt darin, neben den in Veröffentlichungen, Akten und anderen Dokumenten notierten Informationen einen direkten Zugang zu Perspektiven von Akteur*innen zu erhalten, die in klassischen schriftlichen Quellen nicht festgehalten sind. Die historische Forschung erhält die Möglichkeit, mündliche Überlieferungen, Meinungen, Einstellungen, Ereignissen oder Erfahrungen zu erheben und zu analysieren. [7] Werden die Interviews neben schriftliche Quellen (und wo vorhanden auch Bild-, Film- und andere Tondokumente sowie Objekte als historische Quellen) gestellt, kann das Bild der professionellen Etablierung, fachlichen und technischen Entwicklungen sowie der sozialen Einbettung der deutschen Humangenetik in der Nachkriegszeit um Wahrnehmungen und Emotionsschilderung einzelner Akteur*innen ergänzt werden. Daneben kann dieses Bild auch in Gegenwart und Zukunft Anlass zur kritischen Reflektion sozialer, medialer, politischer, medizinwissenschaftlicher und rechtlicher Perspektiven auf die Humangenetik sein.

Die biographische Erzählung ist als eine „historische Narration“ zu verstehen und nicht als „originale“ vergangene Erfahrung. Den Historiker*innen begegnen die „Geschichten“ der Interviewpartner*innen, die ihre Erfahrungen in historische Zusammenhänge einordnen und interpretieren. [8] Sinnbildungen und Deutungen zu zurückliegenden Prozessen und Ereignissen können unterschiedliche Akzente setzen, also einzelne Aspekte hervorheben, andere zurücknehmen oder gar weglassen. Ein Spezifikum der Oral History als historische Methode ist, dass die Forschenden an der Produktion der Quellen unmittelbar beteiligt sind und die generierten Daten ein Ergebnis individueller Interaktionsprozesse sind. [9] Dies bedeutet, dass sowohl die Gesprächspartner als auch die Forschenden das historische Narrativ beeinflussen können.

## Methode der Datenerhebung

In der qualitativen Sozialforschung existiert eine Vielzahl verschiedener Auswahlverfahren zur Ziehung von Stichproben. Im vorliegenden Projekt war zur Auswahl des Samples eine theoretische Stichprobe gezogen worden, um verschiedene Perspektiven professioneller Akteur*innen mit unterschiedlicher Prominenz, fachlicher Ausrichtung und biographischer Zugehörigkeit, vergleichbarer zu machen. Die Verfügbarkeit von lebenden Zeitzeug*innen und die Bereitschaft zur Teilnahme reduzierten die theoretische Stichprobe auf ein so genanntes Convenience-Sampling. Dies ist eine Form eines nicht-zufälligen Auswahlverfahrens. Dies bedeutet, es wird an Hand von einfacher Verfügbarkeit eine nicht repräsentative Stichprobenauswahl getroffen.

Die resultierende Gruppe ist nur bedingt geeignet, daraus allgemeingültige Aussagen abzuleiten, es lassen sich aber durchaus Generalisierungen ableiten, wenn sich beispielsweise in Bezug auf bestimmte Narrative Sättigungseffekte ergeben. [10] Theoretische Sättigung bedeutet, dass im Datenmaterial durch die Hinzufügung weiterer Aussagen kaum neue, relevante Informationen ergänzt werden und somit verallgemeinerbare Aussagen gerechtfertigt erscheinen. [11], [12], [13], [14]

## Beschreibung der Stichprobe

Entgegen der ursprünglichen Planung von 15–25 Interviews wurden schließlich 33 Interviews geführt, da die interviewten Personen weitere, potentiell relevante Gesprächspartner*innen benannten, die dann ebenfalls befragt werden konnten. Die Auswahl beinhaltet 20 Akteur*innen der Humangenetik, die vom Arbeitskreis „Oral History“ der GfH vorgeschlagen wurden. Diese Personengruppe wurde von den beauftragten Historikern zu einem Sample erweitert, das sowohl Geschlecht, Alterskohorten, regionale als auch fachliche Zuordnung berücksichtigte.

Es wurden Träger relevanter Merkmalskombinationen zu einem sog. theoretischen Sample zusammengestellt. [4] Insgesamt wurden 25 Männer und 8 Frauen aus den Geburtsjahrgängen von 1925 bis 1950 interviewt. Nach dem Abschluss und der Freigabe der Interviews konnten 29 Interviewaufzeichnungen ausgewertet werden. 24 Westdeutsche, 2 Ostdeutsche, 2 Österreicher und 1 Schweizer hatten ihre Daten zur Auswertung freigegeben. Alle Interviewpartner*innen waren Professor*innen an Universitäten oder Hochschulen. Ihre Ausbildung beschrieben sie wie folgt (eine Nennung pro Interview): Medizin (21) und Naturwissenschaften (8). Auf die Frage, auf welchem Weg sie in Humangenetik kamen, nannten die Interviewpartner*innen (Mehrfachnennungen möglich, in nicht allen Interviews wurde diese Frage beantwortet): Medizin (12), Pädiatrie (einschließlich Perinatologie) (7), Biologie (einschließlich Botanik) (4), Biochemie (2), Gerichtsmedizin (1), Innere Medizin (1).

Die Nicht-Teilnahme (nonresponse) an einer Erhebung stellt ein zentrales Problem in der empirischen Sozialforschung dar. [15] Hierfür gibt es verschiedene Gründe: 1. die vorgesehenen Person kann nicht kontaktiert werden, 2. Der Person fehlen die Fähigkeit und/oder die Bereitschaft zur Teilnahme und 3. das Einverständnis, die erhobenen Daten zur Verwendung freizugeben, kann nicht erhoben werden. Grundsätzlich lässt sich die Gruppe der Nonresponenten nicht als homogene Gruppe verstehen. Bezogen auf den hier betrachteten Personenkreis ist die Forderung nach einer fallbezogenen Begründung für eine Nichtteilnahme aufgrund der zugesicherten Anonymität und des Datenschutzkonzepts forschungsethisch nicht vertretbar. [6]

## Datenerhebung

Die biographischen Gespräche wurden von der Historikerin Felicitas Söhner geführt. Die Interviews wurden durch einen Leitfaden strukturiert, trotzdem wurde darauf Wert gelegt, die Befragten möglichst frei sprechen zu lassen, um über ihre Erzählung eine Quelle zu produzieren. [4] Durch die direkte Beteiligung (durch Auswahl der Gesprächspartner, Fragestellungen, Anwesenheit, Kommunikation mit den Interviewten) spielen die Interviewenden eine aktive Rolle in der Produktion der Quelle; dies muss in die Quellenkritik einbezogen werden. [7]

Der Leitfaden beinhaltete Fragen zu professionellem Hintergrund, Forschungsnetzwerken, persönlichen Idealen und Zielen sowie Verortung der eigenen Rolle, des Faches und fachlicher Institutionen. [4] Gleichzeitig wurde versucht, das Interviewsetting und die Richtung der Antworten möglichst wenig zu beeinflussen. Die Fragen wurden offen formuliert, so dass Gesprächspartner*innen die Möglichkeit hatten, den Gesprächsverlauf auf ihnen relevant erscheinende Inhalte zu lenken. Über den erzählgenerierenden Ansatz der Fragen im narrativen Interview lassen sich Verlaufsformen von sozialen Veränderungsprozessen erfassen und Kategorien zu deren weiterer Analyse entwickeln. Die Prozessorientierung des leitfadenorientierten Interviews ermöglichte die Erfassung umfassender Erzählungen, der narrative Fragestil ließ eine Erhebung von Daten im Zusammenhang mit der Fragestellung zu. Die Formulierung der Nachfragen orientierte sich an einem inhaltlichen und zeitlichen Horizont, um individuelle und institutionelle Entwicklungsverläufe zu erfassen. [6], [8], [16], [17]

## Methoden der Datenauswertung

In der Auswertung der Interviews geht es u. a. darum, biographische Berichte zu dekonstruieren, also die Struktur der Narration zu erschließen. Ein Ziel liegt darin, Deutungsmuster und Sinnbildungen herauszuarbeiten. Auch die Frage, welche Wertungen in die Narration einfließen, steht im Mittelpunkt des Forschungsinteresses. Die Auswertung erfolgt nach qualitativen, historisch-hermeneutischen und sozialwissenschaftlichen Methoden. Anwendung finden die Ansätze der qualitativen Inhaltsanalyse nach Mayring [18] und Kuckartz [10] sowie der so genannten Grounded Theory [19].

Die qualitative Inhaltsanalyse nach Mayring fokussiert auf die Ordnung, Kategorisierung und Strukturierung von manifesten und latenten Inhalten und die Entwicklung von systematisch und intersubjektiv überprüfbaren Ergebnissen. [18] Die Grounded Theory verfolgt das Ziel, ausgehend von einer offenen Fragestellung mittels inhaltlicher Analyse von Interviews, Feldbeobachtungen und anderen empirisch erhobenen Daten neue Theorien zu entwickeln. [20] Dabei ist die Grounded Theory weniger als einzelne Methode anzusehen, sondern als ein qualitativer Forschungsstil, der eine Sammlung von Forschungsmethoden und Verfahren zusammenfasst. In diesem Sinne wird sowohl induktiv aus den Daten ein Kategorienschema gebildet und fortlaufend angepasst, als auch deduktiv das Schema auf die Datengrundlage angewendet. Die Gesprächsinhalte werden so kodiert und thematisch in Kategorien klassifiziert und zusammengefasst. Legewie spricht wegen des „Pendeln[s] zwischen Induktion und Deduktion, Datenerhebung und Dateninterpretation“ von einem „Dialogcharakter“ der Methode, der es schließlich erlaubt, bei einer „datenverankerte[n] Theorie“ anzukommen. [21]

Das Ziel der Analyse liegt im Entdecken von Erzähl-mustern und Kategorien in den erhobenen Daten und dem Bilden von historischen Hypothesen und neuen Erklärungsansätzen im Sinne eines hermeneutischen Zirkels. Dies bedeutet, der Auswertung liegt ein Vorverständnis zu Grunde. Die Analyse führt zu einer Erweiterung des ursprünglichen Vorwissens. Mit weiteren Durchgängen der Quellenanalyse entwickelt sich ein fortschreitendes Verständnis der Inhalte. [10]

Zur Datenauswertung in der Grounded Theory stehen verschiedene Instrumente zur Verfügung. [10], [18], [20], [22] In diesem Projekt wurde die Einteilung und Strukturierung der Kategorien nach Kuckartz vorgenommen. [10] Da die Anwendung von Gütekriterien quantitativer Analysen auf die qualitative Forschung kontrovers diskutiert wird, [18], [22] haben wir uns entschlossen, wie von Kuckartz [10] vorgeschlagen, alternative Maße interner und externer Studiengüte einzusetzen. Die Gütekriterien der internen Studienqualität (Zuverlässigkeit, Glaubwürdigkeit, Verlässlichkeit) werden durch transparente Darstellung und Dokumentation des Forschungsprozesses erreicht. [10] Zur Einhaltung der externen Gütekriterien (Übertragbarkeit und Verallgemeinerung) wurden nicht direkt in die Befragung involvierte Fachpersonen in die Diskussion der Kategorien einbezogen. [10] Hierzu gehörten u. a. Mitglieder des Arbeitskreises Oral History in der Humangenetik.

## Datenschutz

Die Gesprächspartner*innen der Interviews wurden darüber aufgeklärt, dass die Gespräche aufgezeichnet und gespeichert werden. Sie sind auch über den Rahmen und die Intentionen der Forschung aufgeklärt worden. Interviews werden für dieses Forschungsprojekt nur ausgewertet, wenn die Gesprächspartner*innen dem schriftlich zugestimmt haben. Wörtliche Zitate aus Interviews werden den Beteiligten vor Verwendung in Publikationen vorgelegt. Interviewpartner*innen haben die Möglichkeit, der wörtlichen Verwendung ohne die Nennung von Gründen zu widersprechen. Die Interviews werden für Auswertungen pseudonymisiert und nur bei historischer Notwendigkeit (Kontextualisierung, Zuordnung weiterer Quellen) für den Auswertungsprozess entschlüsselt.

## Dokumentation und Archivierung

Die zur wissenschaftlichen Verwendung freigegebenen Tondokumente wurden digital archiviert. Die Tonaufnahmen selbst stellen die historischen Primärquellen dar. Als Findmittel wurde zu jedem Interview ein Regest erstellt. Die Regesten enthalten Informationen zu Ort und Zeitpunkt des Interviews, zu den beteiligten Gesprächspartnern, technische Daten zur Aufzeichnung und Hinweise auf Zugangsbeschränkungen. Ihren Kern bildet die Information, zu welchem Zeitpunkt im Interview welcher Fragenkomplex besprochen wird. Die Regesten enthalten dazu Stichworte aus den Antworten der Gesprächspartner*innen. Für zukünftige Veröffentlichungen werden Abschnitte der Interviews transkribiert, wenn ein*e Befragte*r im Wortlaut wiedergegeben werden soll.

## Ergebnisse

Die Gesprächsinhalte lassen sich folgenden Kategorien und Sub-Kategorien zuordnen:

(1) biographische Daten und Werdegang der Gesprächspartner*innen (sozialer und Bildungshintergrund; Motivation für den Weg in die Humangenetik; Mentoren und wissenschaftliche Vorbilder; Wahrnehmung der eigenen Rolle im Fach sowie besonderer Leistungen und Schwierigkeiten),
(2) Entwicklung und Anwendung von diagnostischen und therapeutischen Techniken (Chromosomenanalyse; Amniozentese; Molekulargenetik; Epigenetik)
(3) Etablierung und Ausbau der Institutionen der Humangenetik (fachliches Profil; Beratungsstellen; Gründung der GfH; Regelung der Ausbildung und Facharztanerkennung; internationale Kontakte), sowie
(4) gesellschaftliche Einbettung des Faches in Deutschland (Wissenschaftskommunikation mit Politik, Medien und Öffentlichkeit; Dialog mit Patientenorganisationen und Organisationen von Menschen mit Behinderungen; Historische Verantwortung vor dem Hintergrund der Eugenik in Deutschland; Attitüden gegenüber in die Medizin im Nationalsozialismus oder Eugenik verstrickten Akteur*innen).

Entlang dieser Kategorien kann in folgenden Studien ein Vergleich mit anderem Quellenmaterial ebenso erfolgen wie eine heuristische Annäherung an die Gesprächsinhalte zur Entwicklung neuer Fragestellungen zur Geschichte der Humangenetik in Deutschland.

Zugänglich für Forschende sind die Interviews im Archiv der GfH in München. Nach dem Abschluss und Freigabe der Interviews sind 29 Interviewaufzeichnungen mit Regesten 2018 an die GfH übergeben worden. Die Aufzeichnungen der Interviews sollen langfristig als historische Quellen erhalten werden, die zukünftig unter verschiedenen Aspekten ausgewertet werden können. Die GfH bewahrt damit einen Teil ihrer fachkulturellen Erinnerung. [23], [24], [25]

## Diskussion

Die Methode der Oral History bietet die Möglichkeit, historische Perspektiven zu untersuchen, die über schriftliche Dokumente nicht ohne weiteres zu erheben sind. Dieser Zugang weist Spezifika auf, die ihn als nützliches, wenn auch nicht gänzlich problemloses zeithistorisches Instrument ausweisen. Denn die Weitergabe des Erlebten bildet keine historischen Fakten ab, sondern ist in der Narration bereits verarbeitetes Wissen: [26] so ist Subjektivität ein zentraler Aspekt der Oral History. [27] Mit dieser Methode lassen sich historische Narrative als Verarbeitung subjektiver Vergangenheit rekonstruieren, die dann auf Abweichungen vom bisherigen Quellenstand untersucht werden können. [16]

Gleichzeitig bieten biographische Interviews neben einem Mosaik von Erinnerungen „eine zusätzliche Orientierungshilfe“. [28] Sie helfen Quellen einzuordnen, neue Quellen zu identifizieren, Entwicklungen zu verstehen und heuristische Raster zu justieren. In der Oral History können zudem auch Personen zu Wort kommen, die keine oder wenige Schriftquellen hinterlassen haben. So hat die Oral History das Potenzial, die Zeitgeschichte mit bisher „stillen“ Akteur*innen anzureichern.

Die Auswertung der bisher geführten Interviews mit Zeitzeug*innen der Humangenetik unterliegt gewissen inhaltlichen und methodischen Einschränkungen. Eine der Limitationen stellen die Samplingstrategie und die Stichprobe selbst dar. Die Auswahl der in diesem Projekt Befragten erfolgte nach den Kriterien des Convenience Sampling. Die Gesprächspartner*innen wurden nach ihrer Verfügbarkeit, Bereitschaft und Fähigkeit zur Information so ausgewählt, dass die Breite des Gebiets möglichst abgedeckt wurde. Liegt eine Stärke dieser Variante des Samplings darin, dass die Befragten zugänglich und die Durchführung der Erhebung kosten- und zeitgünstig realisierbar ist, so ist kritisch zu hinterfragen, wie weit sich über diese Form des Sampling repräsentative Ergebnisse erzielen lassen. [29] Um möglichen Limitationen des Convenience Sampling zu begegnen, wurde auf breite Heterogenität der Fälle geachtet [4], [30]. Dennoch kann nicht ausgeschlossen werden, dass eine abweichende Stichprobenauswahl zu anders gelagerten Ergebnissen geführt hätte. Für die kleine Gemeinschaft der deutschsprachigen Humangenetiker*innen kann aber nach Meinung der Autor*innen eine Generalisierbarkeit der gesammelten Narrative angenommen werden. Im Vordergrund möglicher Auswertungen steht außerdem aufgrund der Komplexität der Lebensläufe [16] auch weniger das Interesse an abstrakter Objektivität, [7] sondern das Bemühen, aus der Analyse von Einzelfällen deutliche Tendenzen herauszuarbeiten [31] und Geschichte aus Sicht von Teilnehmer*innen zu verstehen. [17], [32]

Dem Anspruch auf Verallgemeinerbarkeit wird nicht zuletzt über eine „theoretische Sättigung“ begegnet, verbunden mit dem Ziel, durch das Sample der Gesprächspartner*innen ein verkleinertes Abbild der Grundgesamtheit zu erhalten. [33]

Über den vorab entwickelten Gesprächsleitfaden [4] ließ sich sicherstellen, dass relevante Befragungsbereiche in allen Interviews enthalten waren. Leitfadengestützte Interview sind zum einen offen genug, um neue qualitative Daten zu generieren, doch andererseits regelgeleitet und systematisch genug, um Forschungsfragen in einer vergleichenden Analyse zu erschließen.

Die Kodierung der Daten erfolgte prozessorientiert und auf Grundlage der empirischen Daten. Da rein deduktive Verfahren in der qualitativen Forschungspraxis als eher nachrangig gelten, [18] wurden die inhaltlichen Kategorien in einem gemischt deduktiv-induktiven Verfahren erstellt. [34] Die Ergänzung durch induktive Kategorien war sinnvoll, um die über Vorwissen gebildeten Kategorien zum Untersuchungsfeld zu ergänzen. [10] Die aus den Interviews heraus gebildeten Kategorien liegen häufig näher an der sozialen Realität, als dies bei theoretisch vorformulierten Hypothesen der Fall sein kann. [24]

Entlang der Kategorien konnte ein Vergleich mit anderem Quellenmaterial ebenso erfolgen wie eine heuristische Annäherung an die Gesprächsinhalte zur Entwicklung neuer Fragestellungen zur Geschichte der Humangenetik in Deutschland. Der Gefahr einer eventuellen persönlichen Voreingenommenheit wurde dadurch ein Gegengewicht gesetzt, dass diese Frage methodisch reflektiert und die Daten von verschiedenen Wissenschaftler*innen interpretiert wurden. Eine Objektivierung des Verfahrens erfolgte über die Ausarbeitung des Leitfadens sowie durch die systematische Datenanalyse durch ein regelgeleitetes Vorgehen. [10], [18]

Die Dateninterpretation basierte auf repräsentativen Gesprächssequenzen als Analyseeinheiten nach den Regeln der qualitativen Inhaltsanalyse [10], [18]. Daraus generierte Ergebnisse wurden mit anderen historischen Quellen verglichen. Die Analyse sowie die Interpretation der Befunde wurden kommunikativ validiert. [6]

## Ausblick

Auf Basis der biographischen Interviews können zukünftig etwa Fragen zu **(1)** biographischen Daten und Werdegang der Gesprächspartner*innen, **(2)** Entwicklung und Anwendung von diagnostischen und therapeutischen Techniken, **(3)** Etablierung und Ausbau der Institutionen der Humangenetik sowie **(4)** gesellschaftlicher Einbettung des Faches in Deutschland beantwortet werden.

Aus dem vorliegenden Material (Interviews, Regesten, Kategorienauswertungen, Objektivierung durch weiteres Quellenmaterial) lassen sich u. a. folgende konkrete Fragestellungen ableiten:

– Welche Rollen spielen Geschichtsbewusstsein und historische Verantwortung in der deutschen Humangenetik?
– Welche Rolle spielen ethische Fragen in der humangenetischen Beratungspraxis?
– Wie haben die Entwicklung und Anwendung von diagnostischen und therapeutischen Techniken die Humangenetik geprägt?
– Wie liefen die Prozesse der Trennung der Humangenetik von der Anthropologie und der Gründung der Deutschen Gesellschaft für Humangenetik ab?

Auf Basis des vorliegenden Materials werden die Autor*innen diese Fragen in folgenden Beiträgen adressieren.
